# Qualitative Dynamic Contrast-Enhanced MRI Time–Intensity Curve Analysis for Differentiating Non-Progressive Treatment-Related Changes from Tumor Progression After Stereotactic Radiotherapy for Brain Metastases

**DOI:** 10.3390/cancers18203344

**Published:** 2026-10-16

**Authors:** Andrea Romano, Giulia Moltoni, Anna Maria Ascolese, Emanuela Parri, Cristiana Monopoli, Paolo Matteucci, Carlo Greco, Lucrezia Toppi, Sara Reina, Sara Ramella, Alessandro Bozzao

**Affiliations:** 1 Department of Neuroscience, Mental Health and Sensory Organs—NESMOS, Sapienza University of Rome, 00189 Rome, Italy; 2 Neuroradiology Department, Azienda Ospedaliera Sant’Andrea, 00189 Rome, Italy; 3 Neuroradiology Department, Azienda Ospedaliera San Camillo-Forlanini, 00152 Rome, Italy; 4 Radiotherapy Department, Azienda Ospedaliera Sant’Andrea, 00189 Rome, Italy; 5 Local Sanitary Hospital Rome 5, 00019 Tivoli, Italy; 6 Radiology Department, G.B. Grassi, 00192 Ostia, Italy; 7 Radiotherapy Department, Campus Bio Medico University Hospital, 00128 Rome, Italy; 8 Campus Bio Medico University Hospital, 00128 Rome, Italy

**Keywords:** dynamic contrast-enhanced MRI, time–intensity curves, non-progressive treatment-related changes, tumor progression, brain metastases, stereotactic radiotherapy

## Abstract

**Background**: This exploratory study assessed whether qualitative dynamic contrast-enhanced MRI (DCE-MRI) time–intensity curves (TICs) differentiate non-progressive treatment-related changes (NPTRC) from tumor progression after stereotactic radiotherapy for brain metastases. **Methods**: Thirty-two patients with 51 treated lesions underwent DCE-MRI. Five neuroradiologists independently delineated a volume of interest and classified the resulting TIC as Type A–D. Longitudinal clinical and MRI follow-up provided the reference outcome, which was recorded before the TIC framework was finalized. An A/B versus C/D grouping was evaluated as a secondary exploratory analysis. **Results**: Thirty-five lesions were classified as NPTRC and 16 as tumor progression. All 17 Type A lesions were NPTRC, whereas all 13 Type C/D lesions showed progression. Type B was observed in 21 of 51 lesions (41.2%), including 18 NPTRC and 3 progressive lesions, representing a substantial indeterminate subgroup. The exploratory A/B versus C/D grouping yielded 81.3% sensitivity and 100% specificity. The ordinal TIC analysis yielded an AUC of 0.952. Interobserver agreement was high within the present reader group (Fleiss’ κ = 0.889). **Conclusions**: Type A was associated with NPTRC, Types C/D were associated with progression, and Type B represented an indeterminate pattern. These descriptive findings require independent validation before clinical use.

## Simple Summary

Distinguishing non-progressive treatment-related changes from tumor progression after stereotactic radiotherapy for brain metastases remains a major diagnostic challenge. In this prospective exploratory study, qualitative dynamic contrast-enhanced MRI time–intensity curves were classified into four kinetic patterns (Types A–D) and compared with subsequent lesion evolution during longitudinal clinical and MRI follow-up. Type A was consistently associated with non-progressive treatment-related changes, whereas Types C and D were associated with tumor progression. Type B occurred in both outcome groups, representing an overlapping or grey-zone pattern. An exploratory grouping of Types A/B versus C/D showed encouraging diagnostic performance, and high interobserver agreement was observed for TIC classification. These preliminary findings suggest that qualitative TIC analysis may provide useful complementary information for differentiating treatment-related changes from tumor progression, but validation in larger independent multicenter cohorts is required.

## 1. Introduction

Following stereotactic radiotherapy (SRT) for brain metastases (BMs), treated lesions frequently exhibit dynamic radiological changes during follow-up magnetic resonance imaging (MRI). Despite the high efficacy of stereotactic radiosurgery (SRS) and fractionated stereotactic radiotherapy (FSRT), which achieve 1-year local control rates of up to 95% for small lesions and approximately 80% for larger lesions, differentiating tumor progression from non-progressive treatment-related changes (NPTRC) remains a major clinical challenge [1]. Lesion enlargement, typically occurring between 6 weeks and 15 months after treatment, is observed in approximately one-third of patients; however, in 30–75% of these cases, the increase reflects post-treatment effects rather than viable tumor progression [2,3,4].

Accurate discrimination between NPTRC and tumor progression is essential because these conditions require substantially different therapeutic strategies. To address this diagnostic challenge, ^18^F-DOPA positron emission tomography (PET) has emerged as one of the most reliable imaging techniques. By exploiting amino acid uptake through the L-type amino acid transporter-1 (LAT1), ^18^F-DOPA PET provides highly specific information on tumor metabolism, enabling differentiation between NPTRC and recurrent tumor with high diagnostic accuracy [5,6]. Nevertheless, false-positive findings may occur because inflammatory and reparative processes associated with NPTRC may also demonstrate increased amino acid metabolism, thereby mimicking viable neoplastic tissue on ^18^F-DOPA PET [7].

Although molecular imaging has demonstrated high diagnostic performance, MRI remains the cornerstone of routine post-treatment surveillance. Several MRI-based approaches have therefore been proposed to improve the differentiation between NPTRC and tumor progression [3]. Among structural imaging biomarkers, delayed hyperenhancement on contrast-enhanced fluid-attenuated inversion recovery (FLAIR) images [8] and diffusion-weighted imaging (DWI) with apparent diffusion coefficient (ADC) mapping have shown promising results [9,10]. Functional imaging techniques mainly rely on perfusion-weighted imaging (PWI), which provides physiological information on tissue vascularity and hemodynamics [3,11].

The diagnostic performance of PWI in distinguishing NPTRC from tumor progression remains controversial. Dynamic susceptibility contrast (DSC)-MRI is currently the most widely used perfusion technique in clinical practice [11,12]. By measuring T2*-weighted signal changes during the first pass of a gadolinium-based contrast agent, DSC-MRI estimates relative cerebral blood volume (rCBV), an indirect marker of microvascular density. However, its clinical applicability is limited by susceptibility artifacts and reduced spatial resolution, particularly in small cortical or subcortical lesions [12]. Other perfusion techniques include dynamic contrast-enhanced (DCE)-MRI, which evaluates blood–brain barrier permeability, and arterial spin labeling (ASL), a completely non-contrast technique that measures cerebral blood flow using magnetically labeled arterial blood water as an endogenous tracer [13,14]. Although ASL has shown promising results, its diagnostic performance may be reduced in hypo- or hypervascular metastases [15]. Among these techniques, DCE-MRI appears particularly promising because it simultaneously provides information on tissue vascularization, capillary permeability, and extracellular volume [13].

DCE-MRI is a physiological imaging technique that continuously monitors lesion enhancement immediately after intravenous administration of a gadolinium-based contrast agent [16]. The dynamic enhancement pattern reflects tissue vascularization, perfusion, capillary permeability, and interstitial volume. Highly vascularized lesions with increased capillary permeability typically demonstrate rapid and intense enhancement, whereas less vascularized tissues show slower and more progressive contrast accumulation [17]. Analysis of time–intensity curves (TICs) allows characterization of these enhancement kinetics. During the first-pass phase, high tissue perfusion and microvascular density result in rapid signal enhancement, whereas delayed phases reflect contrast diffusion into the interstitial space, producing characteristic patterns such as persistent enhancement, plateau, or washout [18].

Although qualitative TIC analysis has been applied to several oncological conditions [19,20,21,22,23], to the best of our knowledge, no previous study has specifically investigated the diagnostic value of qualitative DCE-MRI TIC analysis for differentiating NPTRC from tumor progression after stereotactic radiotherapy for brain metastases. Therefore, this prospective exploratory study aimed to investigate the association between qualitative TIC patterns and longitudinal lesion outcomes in irradiated brain metastases presenting with new or evolving morphological or signal-intensity changes on follow-up MRI. Lesion outcomes were assessed using a study-specific longitudinal clinical and imaging reference standard informed by the RANO-BM framework.

## 2. Materials and Methods

### 2.1. Study Design and Patient Population

Between July 2021 and February 2025, 32 consecutive adult patients (18 men and 14 women; mean age ± standard deviation, 60 ± 13 years) previously treated with stereotactic radiotherapy (SRT) for brain metastases (BMs) were prospectively enrolled. Patients were included when routine MRI follow-up demonstrated new or evolving imaging abnormalities requiring further evaluation to distinguish tumor progression from non-progressive treatment-related changes (NPTRC). At the time of DCE-MRI, patients were receiving different systemic anticancer treatments according to the histological and molecular characteristics of the primary tumor. These included immune checkpoint inhibitors in 19 patients, molecularly targeted therapies in 8, hormonal therapies in 3, and cytotoxic chemotherapy in 2. Among patients receiving immune checkpoint inhibitors, 10 were treated with pembrolizumab, 4 with nivolumab, and 5 with other immune checkpoint inhibitors. No patient was receiving corticosteroid treatment at the time of DCE-MRI (Table 1). The study protocol was approved by the local Institutional Review Board (Protocol No. 19 SA_2020) and was conducted in accordance with the Declaration of Helsinki and its subsequent amendments. Written informed consent was obtained from all participants before study enrollment.

### 2.2. Inclusion Criteria

Patients were eligible for inclusion if they met all of the following criteria:

1. Previous stereotactic radiotherapy for brain metastases performed at least six months before study enrollment, with dose and fractionation schedules consistent with current European guidelines [24];
2. Presence of at least one contrast-enhancing intracranial lesion measuring ≥10 mm in maximum diameter and demonstrating new or progressive morphological and/or signal-intensity changes on follow-up MRI, for which conventional imaging could not reliably distinguish between tumor progression and NPTRC;
3. Absence of contraindications to gadolinium-based contrast agent administration;
4. Availability of at least nine months of clinical and MRI follow-up data after the DCE-MRI examination, allowing longitudinal assessment of lesion evolution.

### 2.3. MRI Acquisition

All MRI examinations were performed using a 1.5-T clinical MRI scanner (Signa Voyager, GE Healthcare) equipped with a 32-channel head coil. The imaging protocol included conventional anatomical sequences (T1-weighted, T2-weighted, fluid-attenuated inversion recovery [FLAIR], diffusion-weighted imaging with apparent diffusion coefficient [ADC] maps, and post-contrast three-dimensional T1-weighted fast spoiled gradient echo [3D FSPGR]) together with DCE-MRI. DCE-MRI was acquired before, during, and after intravenous administration of a gadolinium-based contrast agent using a three-dimensional radial volumetric interpolated sequence with the following acquisition parameters: repetition time (TR), 4.5 ms; echo time (TE), 1.6 ms; flip angle, 12°; slice thickness, 2.2 mm; field of view (FOV), 24–26 cm; receiver bandwidth, 41.67 kHz; temporal resolution, 3.8 s; number of dynamic phases, 130; and total acquisition time, approximately 8 min. Gadoteric acid was administered intravenously at a dose of 0.2 mL/kg body weight using a power injector at a flow rate of 3.5 mL/s, followed by a saline flush. The contrast-agent bolus was initiated 5 s after the start of the dynamic acquisition. Given the temporal resolution of 3.8 s, one complete precontrast dynamic phase was acquired for baseline assessment.

### 2.4. DCE-MRI Post-Processing and Qualitative TIC Assessment

The five participating neuroradiologists (A.B., A.R., G.M., E.P., and C.M.) had between 5 and 20 years of experience. During this assessment, readers had no access to the recorded outcome labels, clinical information, or follow-up images.

DCE-MRI datasets were processed on the AW Server 3.2 (Ext 4.9) platform (GE Healthcare) using GENIQ software (Ext 4.9). Automatic motion correction was applied to the dynamic acquisition, which was subsequently co-registered with the high-resolution post-contrast three-dimensional T1-weighted images.

Each of the five neuroradiologists independently delineated a three-dimensional volume of interest (VOI) encompassing the enhancing component of each lesion on post-contrast images. Each reader’s VOI was propagated to the dynamic dataset to generate an individual time–intensity curve (TIC), which was subsequently classified independently by the same reader according to the four qualitative kinetic patterns (Types A–D). Each neuroradiologist directly classified the TIC generated from their own independently delineated VOI; therefore, no separate randomization of TICs was performed for classification. Areas of macroscopic necrosis, susceptibility artifacts, and large intralesional vessels were excluded. After completion of the independent assessments, the TIC classifications assigned by the five readers were compared to evaluate interobserver agreement. Cases with discrepant classifications were subsequently reviewed collegially by examining the original reader-generated TICs, without additional VOI delineation or generation of new curves. A consensus TIC category was then established for each discrepant lesion and used for comparison with the longitudinal lesion outcome.

**Figure 1:**
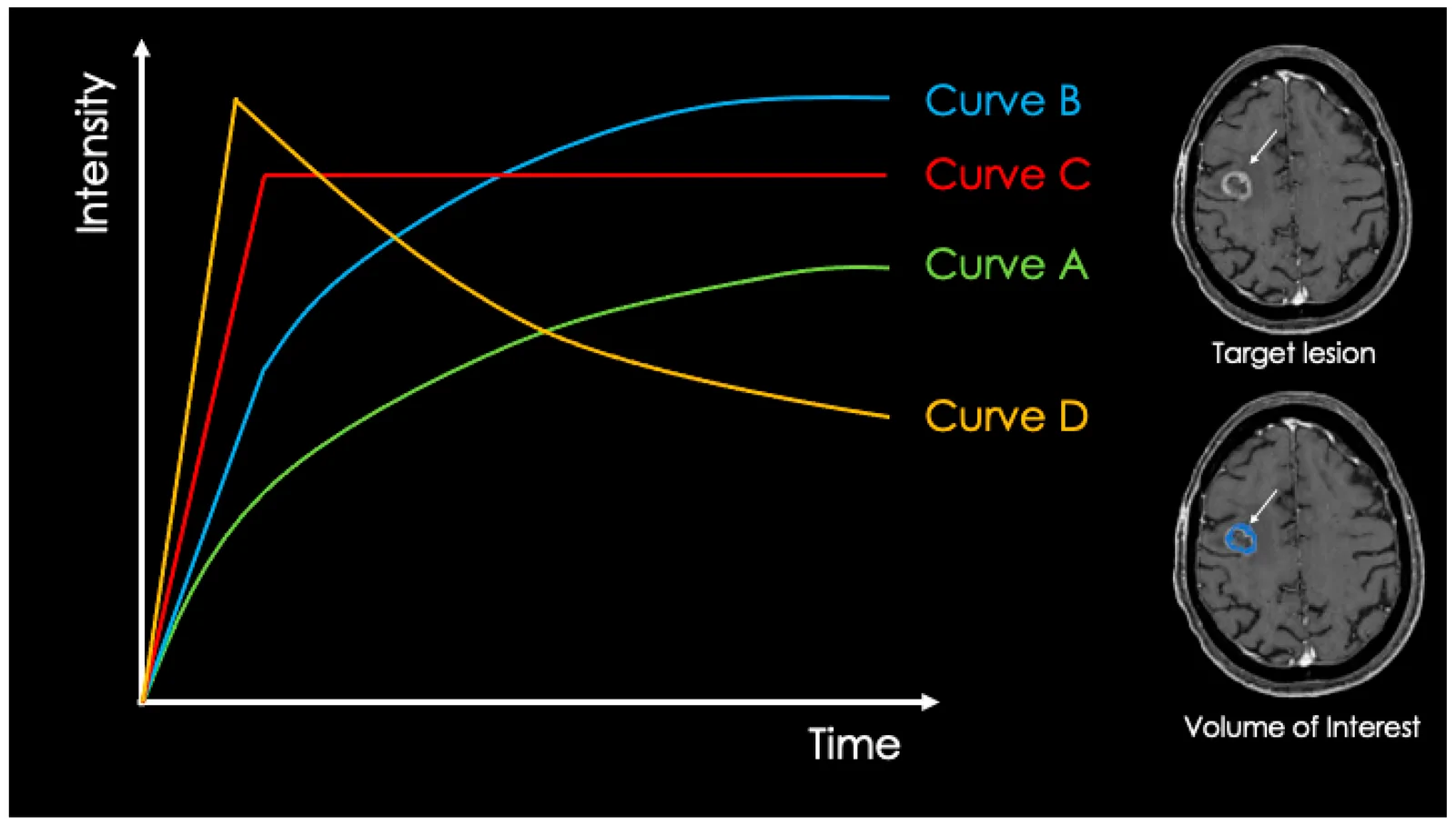
**Representative qualitative DCE-MRI time–intensity curve (TIC) patterns.** Schematic representation of the four qualitative DCE-MRI TIC patterns and lesion-analysis workflow. The upper MR image shows the target enhancing lesion. The lower MR image shows the volumetric VOI manually drawn around the enhancing component of the same lesion. The VOI was propagated to the dynamic dataset to generate the corresponding TIC. The MRI panels are labelled descriptively to avoid confusion with TIC Types A–D.

For each reader-defined VOI, the software automatically generated a TIC displaying raw, non-normalized MRI signal intensity in arbitrary units (a.u.) as a function of elapsed time in milliseconds. No dynamic phases were missing or considered technically inadequate, and no smoothing, fitting, or interpolation procedures were applied to the curves (Figure 1).

Because no established qualitative TIC classification is currently available for irradiated brain metastases, kinetic features previously described in DCE-MRI studies in other oncological settings were adapted to the present clinical setting. Based on curve morphology and the corresponding enhancement kinetics, TICs were classified into four operational patterns (Types A–D):

Type A: Gradual and continuous increase in signal intensity throughout the acquisition, without an identifiable early steep upslope.

Type B: Early rapid signal increase followed by further progressive enhancement during the delayed phase.

Type C: Early rapid signal increase followed by relative signal stability during the delayed phase, without a visually appreciable increase or decrease.

Type D: Early rapid signal increase followed by a visually appreciable decrease during the delayed phase.

Because TIC classification was qualitative, no fixed percentage thresholds were applied.

### 2.5. Longitudinal Outcome Assessment

Longitudinal lesion outcomes were assessed by five neuroradiologists (A.B., A.R., G.M., E.P., and C.M.) based on serial clinical and MRI follow-up. Each neuroradiologist initially evaluated lesion evolution independently, and disagreements or indeterminate cases were subsequently resolved through collegial review of the available follow-up data. Sustained imaging stability or regression was classified as non-progressive treatment-related changes (NPTRC), whereas continued radiological progression was classified as tumor progression. The assessment incorporated longitudinal MRI findings, changes in lesion size, neurological symptoms, corticosteroid use and response, and information on systemic treatments, when available, and was informed by the RANO-BM [25] framework. The final longitudinal outcome categories were established and recorded before the study-specific TIC classification framework was finalized and applied. Subsequently, TIC patterns were assessed without access to the recorded outcome labels, clinical information, or follow-up images. The relationship between TIC categories and longitudinal outcomes was examined only after completion of these assessments.

### 2.6. Comparison Between TIC Patterns and Longitudinal Lesion Outcomes

After completion of the longitudinal follow-up, the TIC pattern assigned at the baseline DCE-MRI examination was compared with the subsequent lesion outcome to investigate whether the different qualitative kinetic patterns (Types A–D) were associated with NPTRC or tumor progression.

As a secondary exploratory analysis, Types A and B were grouped as persistent-enhancement patterns and Types C and D as plateau/washout patterns, and the diagnostic performance of this simplified binary classification was evaluated against the longitudinal outcome.

### 2.7. Statistical Analysis

Interobserver agreement for qualitative TIC classification was assessed using Fleiss’ kappa, which is appropriate for categorical ratings provided by more than two readers. Agreement was calculated for the original four-category classification (Types A–D) and, secondarily, after dichotomization into A/B versus C/D patterns. The proportion of lesions showing complete agreement among all five readers was also calculated. Kappa values were interpreted according to the Landis and Koch classification.

Individual reader classifications were used exclusively for the interobserver agreement analysis. The consensus TIC classification, established after completion of all independent reader assessments, was used for the exploratory analyses of the relationship between TIC morphology and the longitudinally determined lesion outcome.

For the secondary binary A/B versus C/D analysis, tumor progression was considered the positive reference condition and NPTRC the negative reference condition. Sensitivity, specificity, positive predictive value (PPV), and negative predictive value (NPV), together with their exact 95% confidence intervals, were calculated using IBM SPSS Statistics software (version 22).

In a separate exploratory receiver operating characteristic (ROC) analysis, the four TIC categories were treated as an ordinal variable and coded as A = 1, B = 2, C = 3, and D = 4. Tumor progression was defined as the positive reference condition and NPTRC as the negative reference condition. Diagnostic discrimination was expressed as the area under the ROC curve (AUC), with the corresponding 95% confidence interval.

Diagnostic indices and confidence intervals were calculated at the lesion level as descriptive estimates from the cohort in which the TIC framework was developed and initially evaluated. The intervals did not account for the correlation between lesions from the same patient, and the classification was not validated in an independent cohort.

## 3. Results

A total of 51 irradiated brain metastases from 32 patients were included in the analysis. The primary tumor sites were the lung (40 lesions), breast (5 lesions), thyroid (3 lesions), and prostate (3 lesions). During longitudinal clinical and MRI follow-up, 16 lesions (31.4%) were classified as showing tumor progression, whereas 35 lesions (68.6%) were classified as NPTRC.

Complete agreement among all five readers was achieved for 43 of the 51 lesions (84.3%). Disagreements occurred in eight lesions (15.7%) and were exclusively confined to the distinction between Type A and Type B curves. Interobserver agreement for the four-category TIC classification was almost perfect (Fleiss’ κ = 0.889). Following dichotomization into Types A/B versus Types C/D, all five readers agreed on the classification of every lesion (Fleiss’ κ = 1.000). None of the disagreements crossed the A/B versus C/D boundary. The eight lesions with discrepant Type A/B assignments were subsequently reviewed collegially, and a consensus TIC category was established for each lesion.

**Figure 2:**
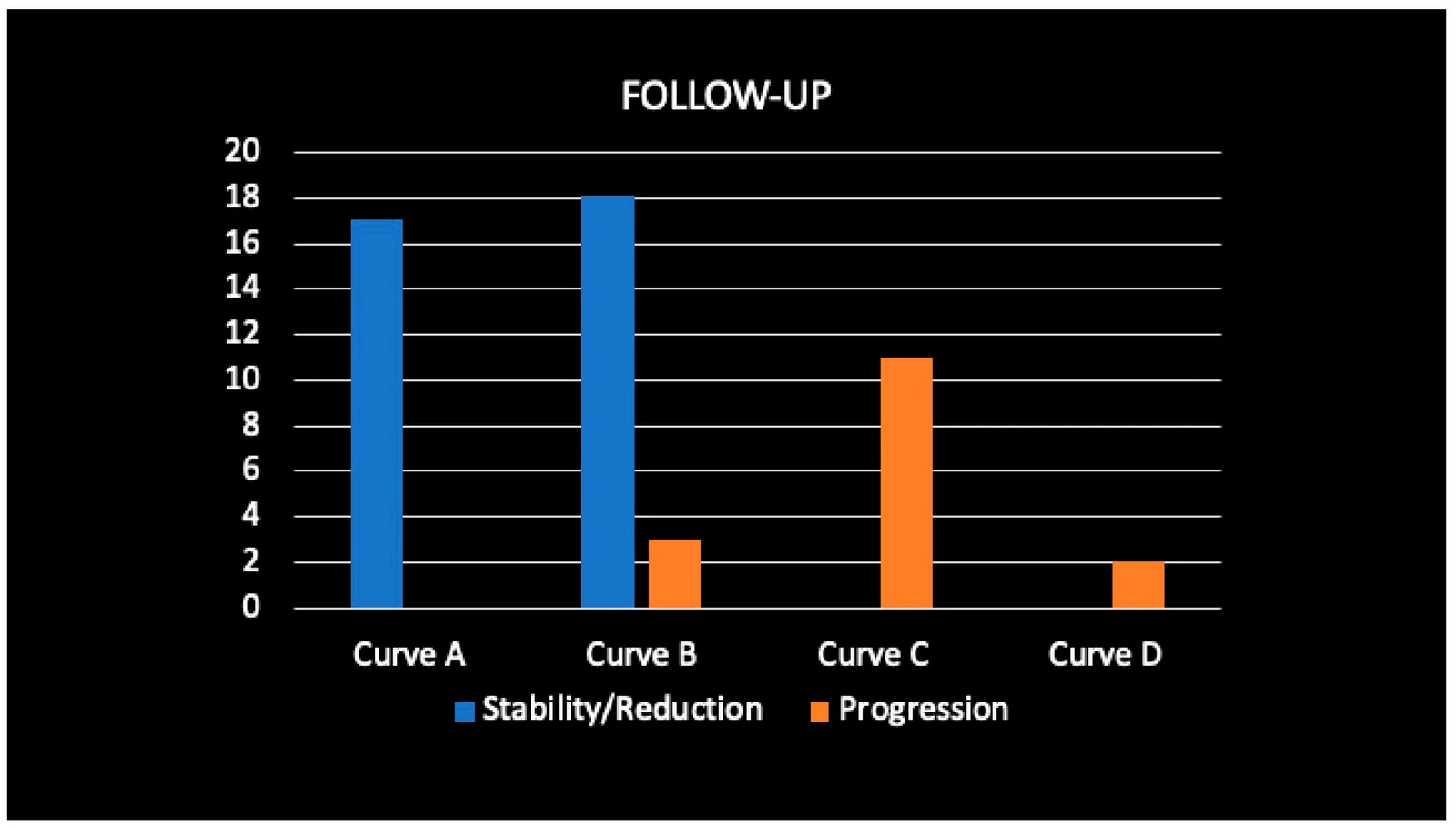
**Distribution of qualitative DCE-MRI time–intensity curve (TIC) patterns according to longitudinal lesion outcomes.** Among the 35 lesions classified as non-progressive treatment-related changes (NPTRC), 17 exhibited Type A curves and 18 exhibited Type B curves. Among the 16 lesions classified as showing tumor progression, 3 exhibited Type B curves, 11 exhibited Type C curves, and 2 exhibited Type D curves. No lesion classified as progressive exhibited a Type A curve.

**Figure 3:**
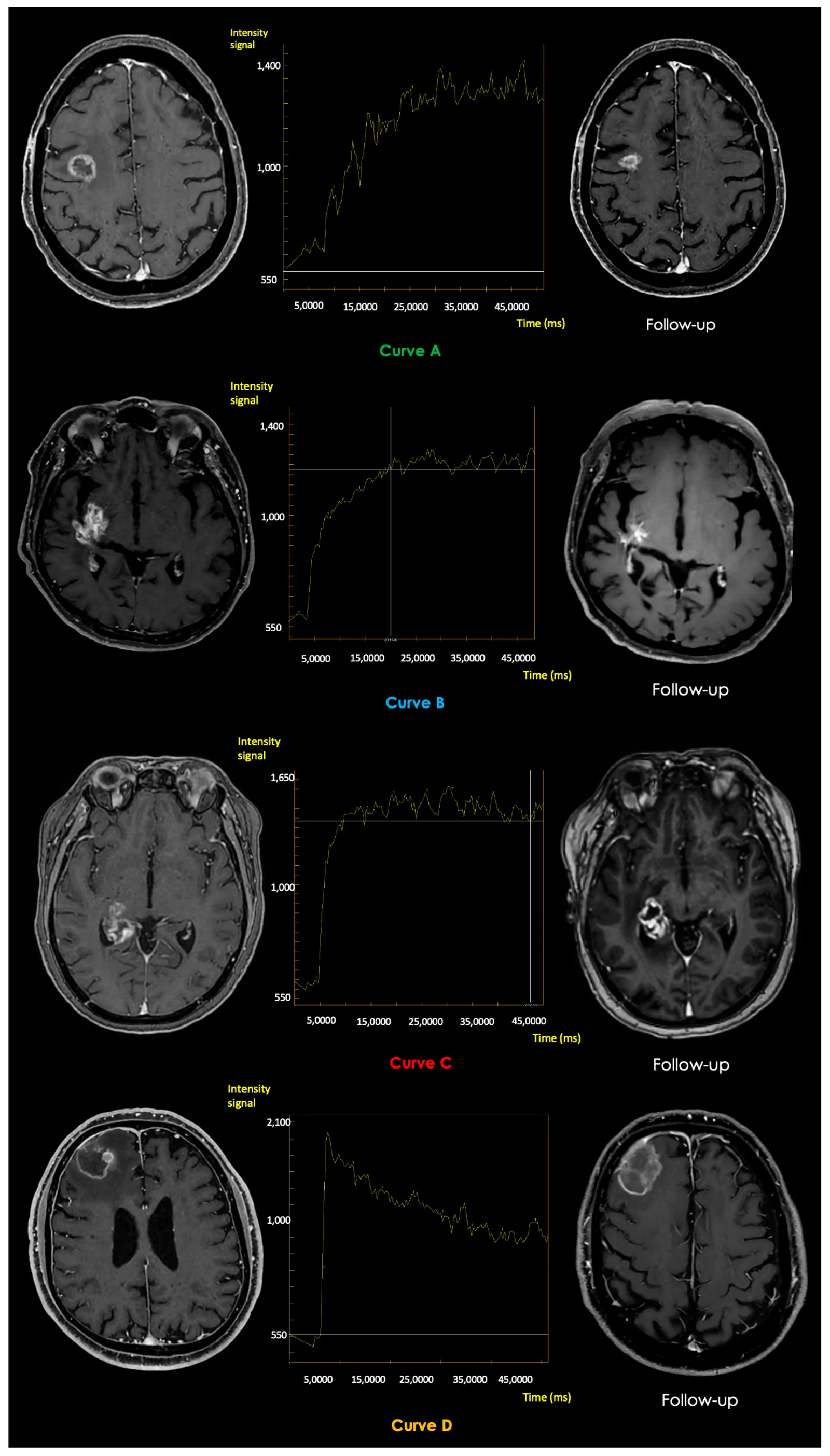
**Representative clinical cases illustrating the four qualitative DCE-MRI time–intensity curve (TIC) patterns.** Each row presents the enhancing lesion at the time of DCE-MRI (left), the corresponding TIC (center), and the lesion appearance at longitudinal follow-up (right). In the present cohort, all lesions exhibiting Type A curves remained stable or decreased in size during follow-up. Type B curves were predominantly associated with NPTRC but were also observed in three progressive lesions, supporting their interpretation as an indeterminate pattern. All lesions exhibiting Type C or D curves showed progressive enlargement during follow-up. Signal intensity is expressed in arbitrary units on the vertical axis.

Comparison of baseline TIC patterns with subsequent longitudinal lesion outcomes showed that, among the 35 lesions classified as NPTRC, 17 (48.6%) exhibited a Type A TIC and 18 (51.4%) exhibited a Type B TIC. Among the 16 lesions classified as showing tumor progression, 11 (68.8%) exhibited a Type C TIC, 2 (12.5%) a Type D TIC, and 3 (18.8%) a Type B TIC. None of the lesions subsequently classified as showing tumor progression exhibited a Type A TIC (Figure 2 and Figure 3).

Type B TICs were observed in 21 of 51 lesions (41.2%), including 18 lesions subsequently classified as NPTRC and 3 showing tumor progression. Among the remaining 30 lesions, all 17 Type A lesions were associated with NPTRC, whereas all 13 Type C/D lesions were associated with tumor progression. Thus, Type A and Type C/D patterns showed complete separation between the two longitudinal outcome groups in the present cohort, whereas Type B represented an overlapping, indeterminate pattern.

As a secondary exploratory analysis, Types A/B were grouped as persistent-enhancement patterns and Types C/D as plateau/washout patterns. This binary classification yielded a sensitivity of 81.3% (95% CI, 54.4–96.0%), specificity of 100% (95% CI, 90.0–100%), PPV of 100% (95% CI, 75.3–100%), and NPV of 92.1% (95% CI, 78.6–98.3%) for identifying tumor progression (Table 2). These lesion-level estimates and confidence intervals are descriptive results obtained from the derivation cohort; the confidence intervals do not account for within-patient correlation.

In a separate exploratory receiver operating characteristic (ROC) analysis, the four TIC categories were treated as an ordinal variable (A = 1, B = 2, C = 3, and D = 4), with tumor progression defined as the positive reference condition. The area under the ROC curve (AUC) was 0.952 (95% CI, 0.888–1.000).

## 4. Discussion

Differentiating tumor progression from non-progressive treatment-related changes (NPTRC) remains one of the major challenges in the imaging follow-up of patients with brain metastases treated with stereotactic radiotherapy. Histopathological studies have demonstrated that recurrent tumor and NPTRC may coexist within the same lesion, resulting in overlapping imaging features and making conventional MRI interpretation particularly difficult [2]. Indeed, both recurrent disease and NPTRC may present with progressive contrast enhancement, increasing mass effect, and surrounding vasogenic edema, thereby limiting the diagnostic accuracy of conventional structural MRI [3,4].

The clinical implications of this distinction are substantial. Patients with NPTRC are generally managed conservatively with close imaging surveillance or medical treatment, whereas evidence of viable tumor may require additional local treatment, surgery, or modification of systemic therapy [26]. Therefore, imaging biomarkers capable of improving the distinction between these two conditions are of considerable clinical value.

In this prospective exploratory study, qualitative DCE-MRI TIC patterns assessed at the baseline examination showed distinct associations with subsequent longitudinal lesion outcomes. Type A patterns were observed exclusively in lesions subsequently classified as NPTRC, whereas Types C and D were observed exclusively in lesions showing tumor progression during follow-up. Type B patterns occurred in both outcome groups, predominantly among lesions classified as NPTRC, suggesting overlapping enhancement kinetics. These findings suggest that qualitative TIC morphology may provide complementary information for characterizing irradiated brain metastases during follow-up.

High interobserver agreement was observed for qualitative TIC classification. Because each reader generated and classified a TIC from an independently delineated VOI, the observed agreement applies to the resulting category assignments and does not isolate agreement in curve interpretation from variability in VOI delineation. Complete agreement among all five readers was achieved in 43 of 51 lesions, and all disagreements were confined to the distinction between Types A and B. After dichotomization into A/B versus C/D patterns, complete agreement was observed because no disagreements crossed the boundary between persistent-enhancement and plateau/washout patterns. These findings suggest that the distinction between persistent-enhancement and plateau/washout patterns was reproducible among the five readers involved in this study. However, the generalizability of this observation remains uncertain, and reproducibility should be further evaluated in independent cohorts involving different readers and institutions.

As a secondary exploratory analysis, Types A and B were grouped as persistent-enhancement patterns, whereas Types C and D were grouped as plateau/washout patterns. This simplified classification yielded a sensitivity of 81.3%, specificity of 100%, positive predictive value of 100%, and negative predictive value of 92.1% for identifying tumor progression. However, these performance estimates should be interpreted cautiously. Although the exploratory A/B versus C/D grouping showed encouraging diagnostic performance in the present cohort, the substantial proportion of Type B lesions (41.2%), together with the limited sample size and the absence of external validation, restricts the immediate clinical applicability of this classification. Its diagnostic performance and reproducibility require further investigation in larger independent multicenter cohorts.

To the best of our knowledge, this is the first study specifically evaluating qualitative DCE-MRI TIC patterns for this clinical indication.

Perfusion-weighted MRI has been extensively investigated as a non-invasive technique for distinguishing recurrent brain metastases from NPTRC, with dynamic susceptibility contrast (DSC)-MRI representing the most widely studied approach [13,27]. Recurrent lesions generally demonstrate increased relative cerebral blood volume (rCBV) because of tumor neoangiogenesis, and several studies have reported sensitivities and specificities exceeding 80% [5,9,12,27,28,29,30,31,32,33,34]. However, DSC-MRI remains affected by susceptibility artifacts, particularly in hemorrhagic lesions or lesions located close to the skull base, and may be technically challenging in small cortical metastases. Furthermore, a recent meta-analysis reported that approximately 5% of examinations are non-diagnostic because of technical limitations [12,28]. Direct comparisons have also suggested that DSC-MRI remains inferior to ^18^F-DOPA PET for differentiating recurrent tumor from NPTRC [5].

Alternative perfusion techniques have also been investigated. Arterial spin labeling (ASL) offers the advantage of avoiding intravenous contrast administration but has shown variable diagnostic performance, particularly in non-hypervascular metastases [35,36]. Quantitative DCE-MRI has demonstrated encouraging specificity for identifying viable tumor; however, its clinical implementation remains limited by the lack of standardized acquisition protocols, heterogeneous post-processing methods, and inconsistent threshold values for pharmacokinetic parameters such as k^trans^ and plasma volume [37,38].

In contrast to quantitative approaches, qualitative evaluation of TICs does not require complex pharmacokinetic modeling and provides a direct representation of lesion enhancement kinetics during dynamic acquisition. TIC morphology reflects the combined effects of tissue vascularization, capillary permeability, and extracellular volume. Lesions characterized by active neoangiogenesis may demonstrate rapid contrast uptake followed by plateau or washout kinetics, corresponding to Types C and D in the present classification, whereas treatment-induced tissue injury may result in persistent progressive enhancement, as observed in Type A, or an early enhancement followed by further delayed signal increase, as observed in Type B [18,39].

The longitudinal evolution observed in our cohort was consistent with this physiological interpretation. All lesions exhibiting a Type A TIC remained stable or regressed during subsequent follow-up, supporting an association between this kinetic pattern and NPTRC. Conversely, all lesions exhibiting Type C or D patterns showed tumor progression. Type B showed less specific behavior: although most Type B lesions had a non-progressive outcome, three subsequently progressed. This overlap suggests that Type B may represent an indeterminate kinetic pattern rather than a specific marker of NPTRC. From a biological perspective, this pattern could reflect residual inflammatory or reparative changes with delayed contrast accumulation, but similar kinetics may also occur in lesions containing viable tumor. Consequently, lesions exhibiting a Type B pattern may warrant closer longitudinal surveillance.

Accordingly, within the present cohort, our findings support a three-level interpretation of qualitative TIC morphology: Type A as an NPTRC-associated pattern, Type B as an indeterminate or grey-zone pattern, and Types C/D as progression-associated patterns. This interpretation may more accurately reflect the observed longitudinal behavior than a simple binary classification. Similar qualitative analyses of TIC morphology have been successfully applied in musculoskeletal, head and neck, breast, and prostate imaging, where enhancement patterns have been associated with tumor aggressiveness and biological behavior [19,20,21,22,23]. Conversely, no previous study has specifically investigated qualitative TIC analysis in irradiated brain metastases. Previous DCE-MRI studies in gliomas have mainly focused on pharmacokinetic parameters and have reported inconsistent results regarding differentiation between tumor progression and NPTRC [39,40]. Our findings therefore suggest that qualitative TIC assessment may represent a complementary approach to quantitative DCE-MRI analysis, potentially providing additional information for the characterization of irradiated brain metastases. However, its clinical utility requires further validation in independent cohorts.

The present study has several limitations. First, the sample size was relatively small and derived from a single institution, with a predominance of lung cancer metastases. The operational TIC categories were developed and initially evaluated in the same cohort. The apparent separation of Type A and Types C/D, as well as the performance of the exploratory A/B versus C/D grouping, may therefore be optimistic and cannot be interpreted as externally validated diagnostic accuracy. Second, longitudinal outcome assessment was based primarily on serial clinical and imaging findings rather than systematic histopathological confirmation. Consequently, slowly progressive or initially asymptomatic recurrent lesions could potentially have been classified as NPTRC during the available follow-up period.

The same five neuroradiologists participated in longitudinal outcome adjudication and subsequently assessed the TICs. Although recorded outcomes, clinical information, and follow-up images were unavailable during TIC assessment, prior participation in adjudication may have permitted recall of individual cases. Thus, the index-test assessment and reference-standard adjudication were not fully independent, and recall or diagnostic review bias cannot be excluded. The TIC results were not used to establish the reference outcomes.

Systemic treatment represents an additional potential source of heterogeneity. A substantial proportion of patients (19/32, 59.4%) were receiving immune checkpoint inhibitors at the time of DCE-MRI, whereas the remaining patients were receiving molecularly targeted therapies, hormonal therapy, or cytotoxic chemotherapy. Immune checkpoint inhibitors may influence post-treatment imaging findings through immune-cell infiltration, inflammatory changes, and increased blood–brain barrier permeability, potentially affecting DCE-MRI enhancement kinetics [7,25]. Previous DCE studies have demonstrated that immune-mediated pseudoprogression generally exhibits lower plasma volume and permeability-related parameters than true tumor progression despite similar contrast enhancement, suggesting that enhancement alone does not necessarily reflect active neovascularization [41]. Likewise, DSC perfusion studies indicate that recurrent tumors usually demonstrate higher relative cerebral blood volume than NPTRC, although overlap may occur because of inflammatory vascular alterations induced by immunotherapy [7]. Amino-acid PET has also shown promising diagnostic performance in this setting, being less affected by inflammatory changes than conventional MRI and FDG-PET, particularly for differentiating pseudoprogression from true progression [42].

Nevertheless, no studies have specifically evaluated the influence of immunotherapy on qualitative DCE-MRI time–intensity curve morphology. Given the limited sample size and heterogeneity of systemic treatments in the present cohort, treatment-specific subgroup analyses were not considered sufficiently powered. Therefore, a possible influence of systemic therapy on qualitative TIC morphology cannot be excluded and should be specifically investigated in larger prospective cohorts.

Finally, the diagnostic performance analysis was performed at the lesion level, although some patients contributed more than one lesion. Lesion-level observations therefore cannot be considered fully statistically independent, and the reported confidence intervals may underestimate uncertainty because within-patient correlation was not explicitly modeled. In addition, the relatively small number of patients and progressive lesions, together with the absence of false-positive findings in the A/B versus C/D analysis, limits the precision and generalizability of the diagnostic performance estimates. In particular, the observed 100% specificity and PPV should not be interpreted as evidence of perfect diagnostic performance. The clinical significance of the Type B pattern also deserves further investigation in larger prospective cohorts. Overall, the present findings should therefore be considered preliminary and require independent multicenter validation before qualitative TIC analysis can be incorporated into routine clinical decision-making.

## 5. Conclusions

In this prospective exploratory study, qualitative DCE-MRI TIC patterns showed distinct associations with longitudinal lesion outcomes after stereotactic radiotherapy for brain metastases. Type A was associated with non-progressive treatment-related changes, Type B represented a substantial indeterminate subgroup, and Types C/D were associated with tumor progression. The secondary exploratory A/B versus C/D grouping demonstrated encouraging diagnostic performance, with high interobserver agreement observed among the five readers involved in this study. These preliminary findings suggest that qualitative TIC analysis may provide complementary information for lesion characterization. However, larger independent multicenter studies, preferably incorporating histopathological or amino-acid PET correlation, are required to validate its diagnostic performance, reproducibility, and potential clinical utility.

## Figures and Tables

**Table 1:** Patient- and lesion-level characteristics of the study cohort.

Characteristics	Values
**PATIENT-LEVEL CHARACTERISTICS**	**32**
**mean age**	60
**sex (F/M)**	14/18
**Number of lesions per patient**	
1 lesion	19
2 lesions	8
3 lesions	4
4 lesions	1
**Concomitant systemic treatment at DCE-MRI**	
***Immune checkpoint inhibitors***	19
Pembrolizumab	10
Nivolumab	4
other	5
***Molecularly targeted therapies***	8
***Hormonal therapies***	3
***Cytotoxic chemotherapy***	2
***No active systemic treatment***	0
**Corticosteroid treatment at DCE-MRI**	0
**Follow-up duration (months)**	
mean	12.2
range	9–16
**LESION-LEVEL CHARACTERISTICS**	**51**
**Primary tumor histology**	
lung	40
breast	5
prostate	3
thyroid	3
**Anatomical location**	
frontal	18
parietal	8
temporal	10
cerebellar	11
occipital	2
brainstem	2
**Enhancing lesion volume at DCE-MRI**	
mean	2.95 cm^3^
range	0.09–12.4 cm^3^
**Time RT-DCE (months)**	
mean	10.3
range	7–15
**Radiosurgical dose (Gy)**	
**Single-fraction SRS, 20–24 Gy**	39
**Fractionated SRT, 27 Gy/3 fractions**	12
**Treated volume (cm^3^)**	
mean	1.98 cm^3^
range	0.05–13.8 cm^3^

**Table 2:** A 2 × 2 contingency table showing the distribution of the exploratory binary TIC classification (Types A/B versus Types C/D) according to longitudinal lesion outcomes. NPTRC, non-progressive treatment-related changes.

	Tumor Progression	NPTRC	Total
**Positive test (C/D)**	13	0	13
**Negative test (A/B)**	3	35	38
**Total**	16	35	51

## Data Availability

The data supporting the findings of this study are not publicly available due to institutional restrictions concerning the confidentiality and protection of patient data.
